# Further refinement of the differentially methylated distant lung-specific *FOXF1* enhancer in a neonate with alveolar capillary dysplasia

**DOI:** 10.1186/s13148-023-01587-6

**Published:** 2023-10-21

**Authors:** Przemyslaw Szafranski, Rijutha P. Garimella, Haresh Mani, Ryan Hartman, Gail Deutsch, Alan Silk, Alan Benheim, Paweł Stankiewicz

**Affiliations:** 1 Department of Molecular and Human Genetics, Baylor College of Medicine, One Baylor Plaza, ABBR-R809, Houston, TX 77030 USA; 2 Department of Pediatrics, Inova LJ Murphy Children’s Hospital, Falls Church, VA USA; 3 Department of Pathology, Inova Fairfax Hospital, Falls Church, VA USA; 4 Inova Department of Genetics, Inova Fairfax Medical Campus, Falls Church, VA USA; 5 University of Washington School of Medicine, Seattle, WA USA; 6 Neonatology, Fairfax Neonatology Associates, Inova Fair Oaks Hospital, Inova LJ Murphy, Children’s Hospital, Fairfax, VA USA; 7 Division of Pediatric Cardiology, Inova LJ Murphy Children’s Hospital, Falls Church, VA USA

**Keywords:** Gene regulation, Tissue-specific enhancer, Differential methylation, ACDMPV, Developmental lung disorders

## Abstract

Heterozygous SNVs or CNV deletions involving the *FOXF1* gene, or its distant enhancer, are causative for 80–90% of cases of alveolar capillary dysplasia with misalignment of pulmonary veins. Recently, we proposed bimodal structure and parental functional dimorphism of the lung-specific *FOXF1* enhancer, with Unit 1 having higher activity on the paternal chr16 and Unit 2 on the maternal chr16. Here, we describe a novel unusually sized pathogenic de novo copy-number variant deletion involving a portion of the *FOXF1* enhancer on maternal chr16 that implies narrowing Unit 2 to an essential ~ 9-kb segment. Using a restrictase-based assay, we found that this enhancer segment is weakly methylated at ApT adenine, with about twice the frequency of methylation on the maternal versus paternal chr16. Our data provide further insight into the *FOXF1* enhancer structure and function.

**Supplementary Information:**

The online version contains supplementary material available at 10.1186/s13148-023-01587-6.

## Introduction

Heterozygous loss of function of the *FOXF1* gene has been found in 80–90% of neonates with histologically verified alveolar capillary dysplasia with misalignment of pulmonary veins (ACDMPV), a lethal lung developmental disorder [1, 2]. Corroboratively, lungs of the *Foxf1*^+/−^ mice recapitulate many features of ACDMPV lungs [3]. *FOXF1* is expressed in mesoderm-derived tissues, including lung mesenchyme, vascular endothelium, and smooth muscle, where it mediates sonic hedgehog signaling from epithelium of the developing alveoli [4]. Progressive respiratory distress experienced by infants with ACDMPV is often accompanied by persistent pulmonary arterial hypertension (PAH) and abnormalities involving other organs [5].

Analyses of 34 ACDMPV-causative overlapping copy-number variant (CNV) deletions, leaving *FOXF1* intact, enabled us to define the distant ~ 60-kb lung-specific *FOXF1* enhancer region mapping ~ 286-kb upstream of the *FOXF1* gene on chromosome 16q24.1 (chr16:86,212,040–86,271,919, hg19) [1, 2, 6]. Recently, we proposed a bimodal structure and parental functional dimorphism of this enhancer, with its Unit 1 having higher activity on the paternal chr16 and Unit 2 on the maternal chr16 [6, 7]. Unit 1 harbors a highly evolutionarily conserved segment and encodes two antisense long noncoding RNAs, whereas Unit 2 features lung-specific histone 3 modifications characteristic for an active enhancer [6, 7]. Interestingly, two regions in Unit 2, DMR1 and DMR2, were found to be hypermethylated on CpG cytosine in some patients with pathogenic single-nucleotide variants (SNVs) in *FOXF1* [8].

Here, we propose further narrowing Unit 2 of the *FOXF1* enhancer and correlate its activity with differential methylation of ApT adenine.

### Patient

The proband (pt 217.3) was a baby girl born at 39 weeks and 4 days of gestation via spontaneous vaginal delivery. The baby initially did well in the Family Centered Care Unit, but failed the Critical Congenital Heart Disease screen at 24 h of life, with pulse oximetry readings of 70% in the right hand and 64% in the right foot. She was transferred to the Neonatal Intensive Care Unit, where she continued to have low O_2_ saturations, but still appeared clinically well. An echocardiogram at 28 h of age demonstrated PPHN with suprasystemic pulmonary arterial pressures and a restrictive PDA with predominantly right-to-left shunting at 1.9 m/s and a small left-to-right low-velocity diastolic shunt. She was transferred to the tertiary care center. The severity of the PPHN and degree of desaturation fluctuated. Ultimately, she developed persistent arterial O_2_ desaturation (PaO_2_ = 33 mmHg) despite maximal medical management and was transferred to the Pediatric Cardiac Intensive Care Unit for ECMO support. At 5 days of age, she was cannulated for veno-arterial (VA) ECMO. She weaned and separated from VA-ECMO on the 5th day (9 days old), and initially tolerated this well, with support including mechanical ventilation, iNO, epoprostenol, and milrinone. Her O_2_ saturations remained stable for the first 24 h, but then became progressively more labile despite maximizing medical therapy.

Histopathological studies were performed on FFPE tissue stained with hematoxylin and eosin. Lung biopsy showed marked immaturity of the parenchyma with simplified distal airspaces and diffuse septal widening. There was evidence of pulmonary arteriopathy with pulmonary artery branches showing significant concentric medial hypertrophy and microangiopathic changes (Additional file 1: Fig. S1a). There was abnormal muscularization of small vessels in the lobule. Septal capillaries were not closely apposed to alveolar walls. There were pulmonary vein branches near bronchovascular bundles (Additional file 1: Fig. S1b) and lymphatic channels were dilated (Additional file 1: Fig. S1c). These histologic features confirmed a diagnosis of ACDMPV.

## Materials and methods

### DNA and RNA isolation

DNA was extracted from proband’s blood and parents’ buccal smear samples using Gentra Puregene Blood Kit (Qiagen, Germantown, MD). DNA from frozen lung autopsy samples from five previously reported ACDMPV cases (pts 60.4, 64.5, 179.3, 180.3, and 205.3) was extracted using DNeasy Blood and Tissue Kit (Qiagen). RNA was isolated from pts 99.3 [9] and 217.3 FFPE lung tissues using Quick-RNA FFPE Kit (Zymo Res., Irvine, CA).

### Chromosomal microarray analysis and DNA sequencing

Array CGH analysis was done using customized chr16q24.1 region-specific 4 × 180 K oligonucleotide microarray (Agilent, Santa Clara, CA) according to manufacturers’ protocol.

Targeted next-generation sequencing (NGS) was done by Invitae (San Francisco, CA) using Illumina technology with ≥ 50 × depth. CNVs were called using an in-house algorithm that determines CNV at each target.

CNV deletion junction was amplified by long-range PCR using LA *Taq* DNApol (Takara Bio., Madison, WI) and primer pair 5′-GACCCTGATCTTGCATGTTCCTCGT-3′/5′-GAAGAATCGCCATCCCAGGTCAACG-3′, directly Sanger sequenced, and aligned with the human genome sequence GRCh37/hg19 using BLAT tool in the UCSC Genome Browser (https://genome.ucsc.edu).

Parental origin of the CNV deletion was determined using informative SNPs amplified for sequencing from the patient’s region of hemizygosity on chr16q24.1 and from corresponding region of parents’ chr16 with a primer pair 5′-TAACCAGAACTCCTCCCTGCCTGAG-3′ / 5′-AAAGCACCTGTTGATGGACTCTGGT-3′.

### DNA methylation analysis

To determine the epigenetic characteristics of the described portion of Unit 2 of the *FOXF1* enhancer, we analyzed the methylation status of cytosine and adenine within the DMRs, hemizygous in ACDMPV patients with paternal (pts 179.3, 180.3 and 205.3) or maternal (pts 60.4 and 64.5) CNV deletions of the enhancer, leaving *FOXF1* intact (Additional file 2: Fig. S2). Genomic DNA (500 ng) was treated overnight at 37 °C with 5 units of *Hha*I or *Mbo*I (NEB, Ipswich, MA) in 25 µl of the rCutSmart buffer (NEB). The activity of *Hha*I is blocked by cytosine methylation (5mC) in CpG context, whereas the activity of *Mbo*I is blocked by methylation at 6N of adenine (m6A) in ApT context or cytosine in CpGs if they partially overlap with the *Mbo*I site. Following the treatment with restrictases, DMR1 and DMR2, both of which contain a single *Hha*II and *MboI* recognition site, were PCR amplified from 50 ng of nuclease treated or untreated DNA in a volume of 25 µl, using primer pairs listed in Additional file 8: Table S1, and LA *Taq* DNApol, applying 25 amplification cycles. In addition, the randomly selected 0.2-kb region, not recognized by *Hha*II or *MboI*, was simultaneously amplified as an internal control. The PCR products were resolved on EtBr-agarose gels and semi-quantified from gel images using ImageJ software (https://imagej.nih.gov). For comparison of methylation frequencies at maternal and paternal allele of DMRs, the ratios of the intensities of the DMR DNA band to the internal control band were used.

### Real-time PCR

Transcript levels of *FOXF1* and its downstream target *TMEM100* [10] were measured by RT-qPCR. RNA samples were converted to cDNA using SuperScript III First-Strand Synthesis System (Invitrogen, Waltham, MA). TaqMan gene expression assays were obtained from Applied Biosystems (Foster City, CA). RT-qPCRs were performed on BioRad CFX Connect Real-Time System using TaqMan Universal PCR Master Mix (Applied Biosystems). For relative quantification of the transcripts/cDNA, the comparative ∆∆Ct method was used. *FOXF1* and *TMEM100* transcript (cDNA) levels were normalized to that of *GAPDH*, and showed as a fold change of an average *FOXF1* or *TMEM100* transcript (cDNA) level from two normal lung autopsy samples.

### Immunostaining

Immunostaining for TMEM100 (1:50, Sigma HPA055936) on cases 99.3 and 217.3 was performed on the Ventana BenchMark Ultra stainer after CC1 antigen retrieval on FFPE 5-µm sections of the lung. After pretreatment on the stainer, slides for FOXF1 immunohistochemistry (1:100, AF4798, R&D Systems) were blocked for one hour at room temperature using 5% normal donkey serum (Jackson Immuno Research Laboratories) in PBS containing 0.3% Triton X-100 and then incubated with primary antibody diluted in blocking buffer overnight at 4 °C. They were visualized with DAB peroxidase substrate after incubation with donkey anti-goat HRP at a dilution of 1:200 in blocking buffer for 1 h at room temperature. Images were captured with a digital camera mounted on a Nikon Eclipse 80i microscope using NIS-Elements Advanced Research Software v4.60.

## Results

### Genomic structure of the CNV deletion region

**Fig. 1:**
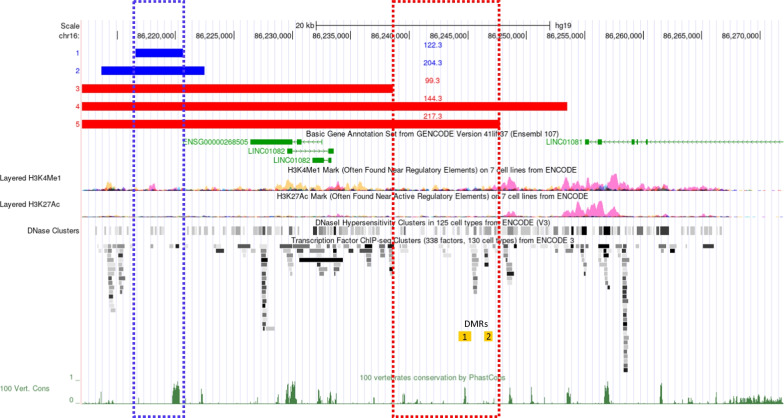
Overview in UCSC genome browser of the *FOXF1* distant enhancer on chr16q24.1. The newly described and other analyzed ACDMPV-causative deletions are shown as red (maternal deletions) and blue (paternal deletions) bars. The essential segments of Unit 1 on the paternal chr16 [7] and Unit 2 on the maternal chr16 are shown with the blue- and red-dotted rectangles, respectively

Targeted NGS and customized aCGH analyses of the proband DNA sample revealed an ~ 46-kb heterozygous CNV deletion at chr16q24.1 (chr16:86,201,444–86,247,738, hg19). The deletion breakpoints mapped to non-repetitive sequences (Additional file 3: Fig. S3a). There was a 3-bp microhomology (CTG) at the deletion junction, suggesting that the deletion arose in a replication-based FoSTeS/MMBIR mechanism. Junction-specific PCR in the parental DNA samples revealed that the deletion arose de novo; there was no evidence of parental somatic mosaicism (Additional file 3: Fig. S3b). Family trio Sanger sequencing of two informative SNPs, rs11640208 and rs11641863, amplified from the region corresponding to the deletion, revealed that the deletion arose on chr16 inherited from the mother (Additional file 4: Fig. S4). The pt 217.3 deletion overlapped 33 other previously reported *FOXF1* enhancer CNV deletions located upstream of *FOXF1* (Additional file 2: Fig. S2), and removed the proximal portion of the 60-kb *FOXF1* enhancer region, including the entire Unit 1 and approximately one third of Unit 2 (Fig. 1). Importantly, this deletion maps within the CNV deletion region (chr16:86,149,407–86,253,509) on maternal chr16 in pt 144.3 who also presented with typical ACDMPV [11] and partially overlaps the CNV deletion region (chr16:86,212,040–86,238,601/86,238,621) on maternal chr16 in pt 99.3 with late onset ACDMPV [9]. These data suggest that an ~ 9-kb portion (chr16:86,238,601–86,247,738) of the pt 217.3 CNV deletion, not included in pt 99.3 deletion, could contain an essential segment of Unit 2 of the enhancer.

We measured transcript levels of *FOXF1* and its target *TMEM100* in lung autopsy specimens from pts 99.3 and 217.3. We found that the deletion in pt 217.3 resulted in a greater decrease of the *FOXF1* expression than in pt 99.3 (*P* < 0.02) (Additional file 5: Fig. S5), further supporting the proposed localization of an essential segment within this ~ 9-kb portion of Unit 2. The *TMEM100* transcript level was substantially reduced in both 217.3 and 99.3 cases, making its correct estimation by RT-qPCR impossible (Additional file 5: Fig. S5). We also compared the expression of FOXF1 and its target TMEM100 in pts 99.3 and 217.3 using antiFOXF1 and antiTMEM100 antibodies. While the expression for FOXF1 and TMEM100 were significantly reduced in both patients, in pt 217.3 it was weaker than in pt 99.3 (Additional file 6: Fig. S6).

### Differential methylation within unit 2

**Fig. 2:**
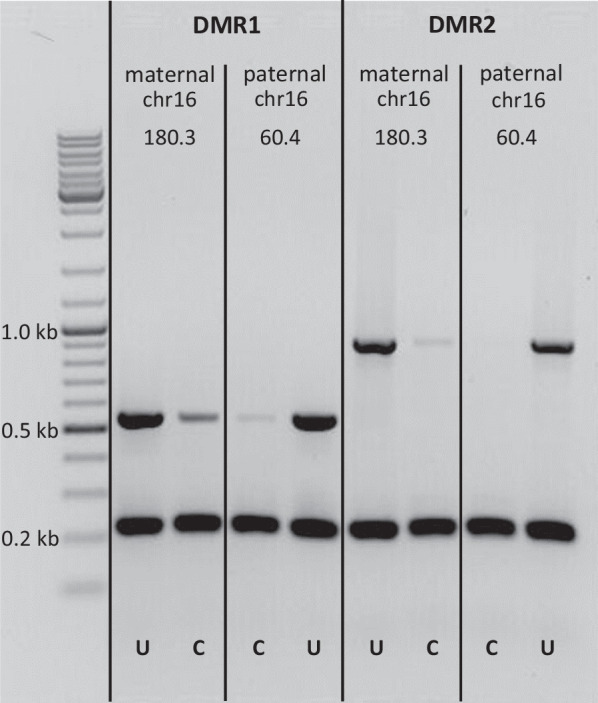
Parent-of-origin-specific differential methylation at N6 of ApT adenine in Unit 2 of the *FOXF1* enhancer. At both DMR regions, PCR amplification from maternal chr16 (paternal deletion), following treatment with *Mbo*I, was about twice stronger, indicating more frequent protection through methylation from endonucleolytic cleavage in comparison to paternal chr16 (maternal deletion). U or C indicates that the DNA used in a given PCR was either untreated or previously treated with *Mbo*I, respectively

We found that DMR1 and DMR2 within Unit 2 were completely protected from methylation-sensitive digestion by *Hha*I, confirming that cytosines in the CpG context in this region of the enhancer were methylated (Additional file 7: Fig. S7). The activity of *Hha*I nuclease was verified by complete digestion with *Hha*I of the PCR-amplified DMRs. Of note, when analyzing DNA with enhancer CNV deletions, we did not find any significant difference between cytosine methylation in this region on maternal versus paternal chr16. Using a similar PCR assay, in which lung DNA was treated with 6 mA-sensitive *Mbo*I, we found that this DNA pool was also, although to much lesser degree (up to ~ 25%), protected from digestion, indicating that a small fraction of lung cells had adenine methylated on N6 in DMR1 and DMR2 (Fig. 2, Additional file 8: Table S2). Importantly, using this assay, we also found that both DMRs were about 1.8 times more often methylated on maternal than on paternal chr16 (15.8 ± 5.3% and 8.8 ± 3.2%, respectively; *P* < 0.05) (Fig. 2, Additional file 8: Table S2). None of the DMR *Mbo*I sites overlapped CpG.

## Discussion

Comparison of the CNV deletion in the proband 217.3 with two previously published overlapping CNV deletions also on maternal chr16, one resulting in severe ACDMPV (pt 144.3) [11] and the other in late onset ACDMPV (pt 99.3) [9], allowed us to identify an ~ 9-kb segment that likely harbors the most essential portion of Unit 2 of the *FOXF1* enhancer. Although the involvement of other genes or noncoding regulatory elemements genome-wide cannot be ruled out, the contribution of the 9-kb enhancer segment seems possible for a number of reasons. This segment harbors binding sites for numerous TFs (ENCODE), including lung-expressed YY1, GABPA, TRIP13, EP300, TRIM22, PLRG1, and RNApol. Of interest, YY1, in addition to functioning as a catalyst for phase-separated droplets of TFs, is thought to enable and stabilize enhancer-promoter interactions [12], whereas EP300 is a histone acetyltransferase involved in H3K27ac modification controlling the accessibility of chromatin within the enhancer for TFs and RNApol [13]. The studied interval of Unit 2 also harbors DNase I hypersensitive sites (ENCODE), indicating its increased accessibility for TFs and RNApol. Importantly, as determined by circular chromatin-conformation-capture (4C), this interval of Unit 2 also contains a lung cell-specific enhancer-promoter contact site (chr16:86,246,725–86,246,982) [6]. This indicates that the TFs binding in this region are in spatial proximity to the *FOXF1* promoter, and thus are likely relevant for its activity (this interaction site is deleted in pts 144.3 and 217.3, but present in 99.3). Corroborating these predictions, the expression of both *FOXF1* and *TMEM100* in pt 217.3 lungs was lower than in pt 99.3, in whom the entire Unit 2 remains intact.

Intriguingly, we also found that DNA of the essential portion of Unit 2 is differentially methylated on adenine at N6 in ApT context. In both maternal and all three paternal deletion cases, 6 mA methylation was on average twice as frequent on the maternally inherited allele of Unit 2 compared to the paternal allele. Higher frequency of adenine methylation on the maternal chr16q24.1 might contribute to the previously suggested higher regulatory activity of Unit 2 on maternal chr16 due to direct specific facilitation of TF binding or reduction of a repressor binding. For example, a *Drosophila* Fox TF Jumu preferentially binds in vitro to 6 mA-methylated DNA [14]. Moreover, adenine methylation at N6 can also alter DNA–protein interaction through general destabilization of double-stranded B-DNA structure [14]. Of interest, two human FOX TFs are known to bind within the discussed essential part of Unit 2 (ENCODE).

In summary, our data indicate that this ~ 9-kb portion of Unit 2 of the *FOXF1* enhancer is likely essential for proper expression of the *FOXF1* gene. This region harbors the enhancer-promoter interaction site and the binding sites for TFs involved in chromatin modification and/or regulation of cell cycle and motility. We also found that this interval is weakly differentially methylated on ApT adenine with on average about twice more frequent methylation of its maternal versus paternal allele. This difference might contribute to the proposed higher enhancement activity of Unit 2 on maternal compared to the paternal chr16.

### Supplementary Information

**Additional file 1: Figure S1.** Lung pathology of the ACDMPV pt 217.3. (**A**) Pulmonary artery branches show medial concentric hypertrophy. One artery shows microangiopathic changes with erythrocytes in vessel wall (H&E, 200x). (**B**) Hypertrophic arteries (arrowheads) in the bronchovascular bundles accompanied by thin-walled shunt vessels (“misaligned pulmonary veins”; arrows) (H&E, 200x). (**C**) Lower magnification demonstrating dilated lymphatic channels (*) and shunt vessels (arrows) (H&E, 100x).

**Additional file 2: Figure S2.** Compilation of ACDMPV-causative CNV deletions at chr16. Deletions that occurred on paternal chr16 are shown as blue bars, those on the maternal chr16 as red bars, and those on chr16 of unknown parental origin as black bars.

**Additional file 3: Figure S3.** (**A**) DNA sequence across the deletion junction. The deletion breakpoints are located within the microhomology region shown on black background. (**B**) De novo origin of the deletion. The deletion junction could only be amplified from the proband’s DNA.

**Additional file 4: Figure S4.** Parental origin of the CNV deletion. Based on SNV segregation, the deletion occurred on the maternal chr16.

**Additional file 5: Figure S5.** Relative *FOXF1* and *TMEM100* transcript levels in lungs with two partially overlapping *FOXF1* enhancer deletions.

**Additional file 6: Figure S6.** Immunostaining of pt 99.3 and 217.3 lung tissues with antiFOXF1 and antiTMEM100 antibodies. Compared to a neonatal control (inserts top panel), there is very limited capillary expression for TMEM100 in pt 99.3 and no expression of TMEM100 in pt 217.3. Both patients showed loss of normal expression for FOXF1 in the arterial endothelium (a). V = shunt veins.

**Additional file 7: Figure S7.** Cytosine methylation within DMR regions of the *FOXF1* enhancer. The frequency of CpG methylation on both maternal and paternal chr16 are similar. U or C indicate that the DNA used in a given PCR either was not or had previously been digested by *HhaI*, respectively.

**Additional file 8: Table S1.** PCR primers used in methylation analysis. **Table S2.** Results of semi-quantitative analysis of DNA protection from *Mbo*I cleavage by adenine methylation in DMR regions of the enhancer Unit 2. The percentage values correspond to the extent of methylation and indicate on average higher methylation of Unit 2 on maternal chr16.

## Data Availability

Not applicable.

## Abbreviations

ACDMPV: Alveolar capillary dysplasia with misalignment of pulmonary veins
aCGH: Array comparative genomic hybridization
CNV: Copy-number variant
DMR: Differentially methylated region
DNApol: DNA polymerase I
ECMO: Extracorporeal membrane oxygenation
FoSTeS: Fork stalling and template switching
FFPE: Formalin-fixed paraffin-embedded
FOXF1: Forkhead box transcription factor 1
iNO: Inhaled nitric oxide
MMBIR: Microhomology-mediated break-induced replication
NGS: Next-generation sequencing
PCR: Polymerase chain reaction
PPHN: Persistent pulmonary hypertension of the newborn
PDA: Patent ductus arteriosus
RNApol: RNA polymerase II
RT-qPCR: Real-time quantitative polymerase chain reaction
SNP: Single-nucleotide polymorphism
SNV: Single-nucleotide variant
TF: Transcription factor
VA-ECMO: Veno-arterial extracorporeal membrane oxygenation
