# Supplementary figures and images for: Further refinement of the differentially methylated distant lung-specific FOXF1 enhancer in a neonate with alveolar capillary dysplasia
Source: Clin Epigenetics. 2023 Oct 21;15:169. doi: 10.1186/s13148-023-01587-6 (PMC10589973; doi:10.1186/s13148-023-01587-6)

## Slide 1
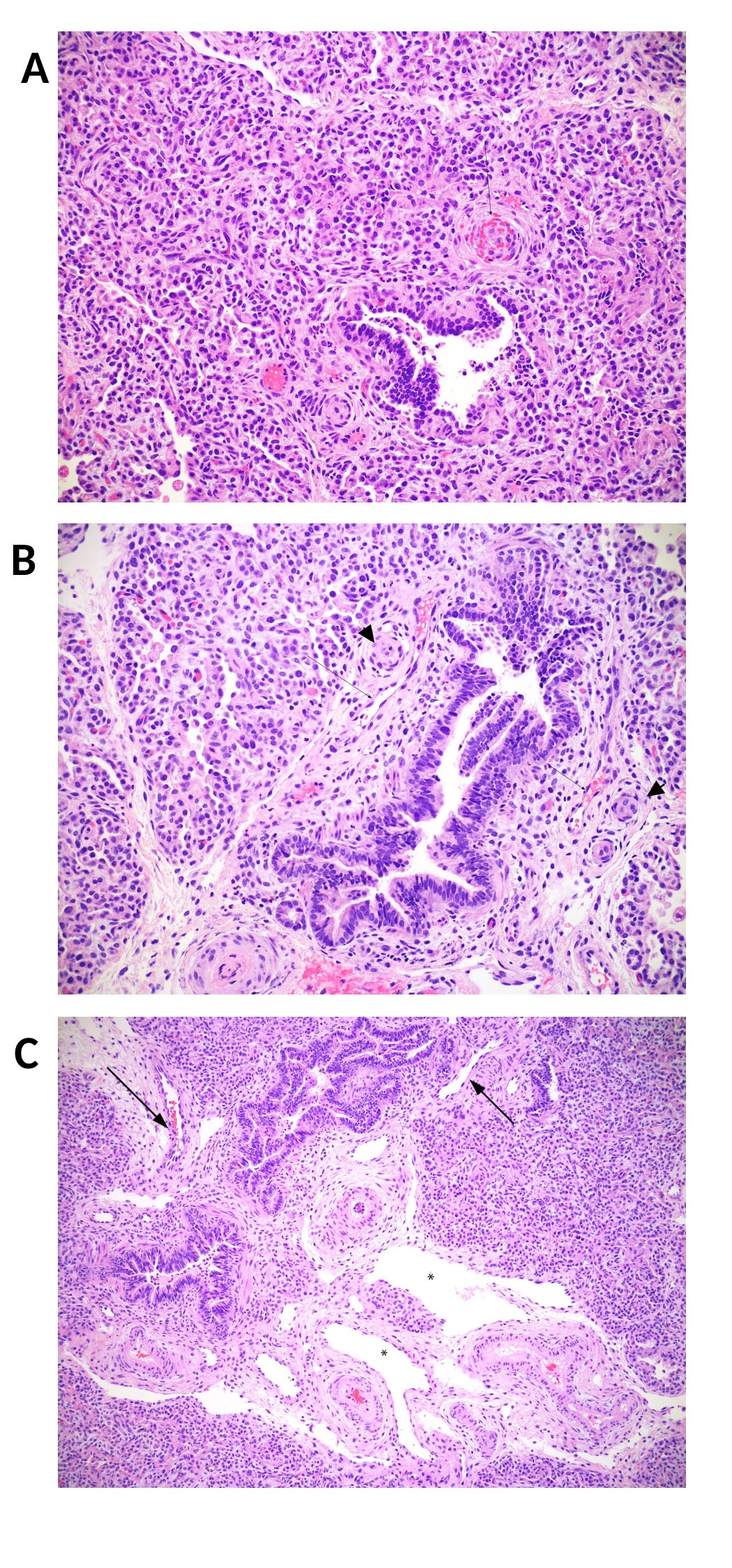

A
B
C

Supplement: Supplementary file 1 — Additional file 1: Figure S1. Lung pathology of the ACDMPV pt 217.3. (A) Pulmonary artery branches show medial concentric hypertrophy. One artery shows microangiopathic changes with erythrocytes in vessel wall (H&E, 200x). (B) Hypertrophic arteries (arrowheads) in the bronchovascular bundles accompanied by thin-walled shunt vessels (“misaligned pulmonary veins”; arrows) (H&E, 200x). (C) Lower magnification demonstrating dilated lymphatic channels (*) and shunt vessels (arrows) (H&E, 100x). [file 13148_2023_1587_MOESM1_ESM.pptx]

## Slide 1
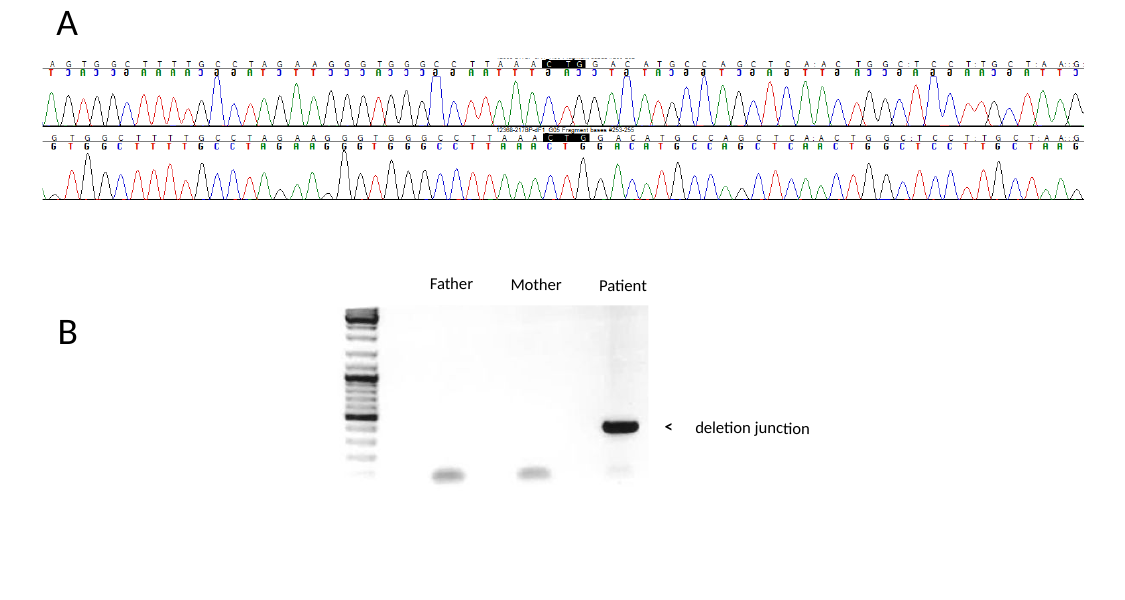

A
Father Mother Patient
< deletion junction
B

Supplement: Supplementary file 3 — Additional file 3: Figure S3. (A) DNA sequence across the deletion junction. The deletion breakpoints are located within the microhomology region shown on black background. (B) De novo origin of the deletion. The deletion junction could only be amplified from the proband’s DNA. [file 13148_2023_1587_MOESM3_ESM.pptx]

## Slide 1
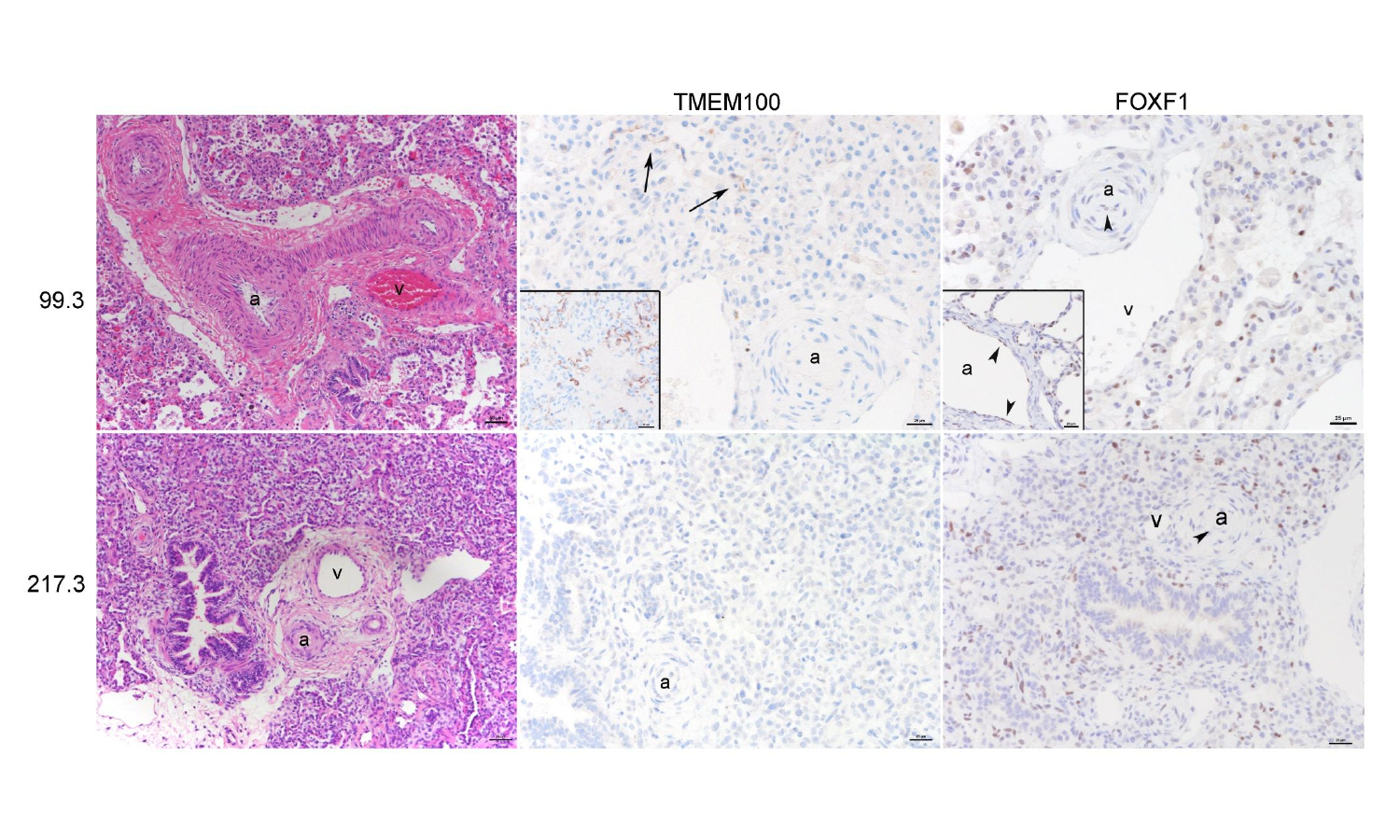

Supplement: Supplementary file 6 — Additional file 6: Figure S6. Immunostaining of pt 99.3 and 217.3 lung tissues with antiFOXF1 and antiTMEM100 antibodies. Compared to a neonatal control (inserts top panel), there is very limited capillary expression for TMEM100 in pt 99.3 and no expression of TMEM100 in pt 217.3. Both patients showed loss of normal expression for FOXF1 in the arterial endothelium (a). V = shunt veins. [file 13148_2023_1587_MOESM6_ESM.pptx]

## Slide 1
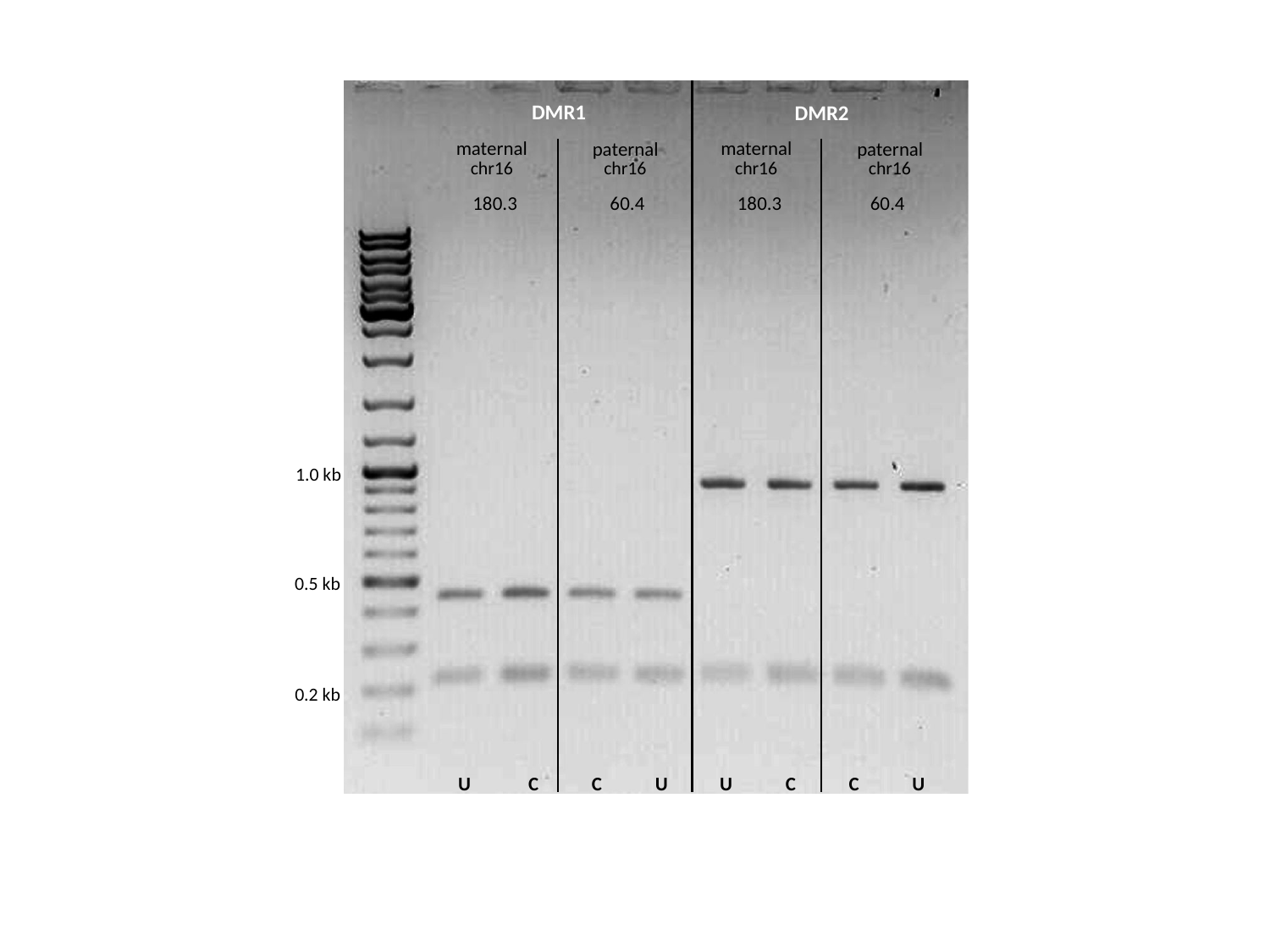

DMR1
DMR2
maternal
maternal
paternal
paternal
chr16
chr16
chr16
chr16
180.3 60.4
180.3 60.4
1.0 kb
0.5 kb
0.2 kb
U C C U
U C C U

Supplement: Supplementary file 7 — Additional file 7: Figure S7. Cytosine methylation within DMR regions of the FOXF1 enhancer. The frequency of CpG methylation on both maternal and paternal chr16 are similar. U or C indicate that the DNA used in a given PCR either was not or had previously been digested by HhaI, respectively. [file 13148_2023_1587_MOESM7_ESM.pptx]
