# Supplementary material for: Further refinement of the differentially methylated distant lung-specific FOXF1 enhancer in a neonate with alveolar capillary dysplasia
Source: Clin Epigenetics. 2023 Oct 21;15:169. doi: 10.1186/s13148-023-01587-6 (PMC10589973; doi:10.1186/s13148-023-01587-6)
Supplement: Supplementary file 2 — Additional file 2: Figure S2. Compilation of ACDMPV-causative CNV deletions at chr16. Deletions that occurred on paternal chr16 are shown as blue bars, those on the maternal chr16 as red bars, and those on chr16 of unknown parental origin as black bars. [file 13148_2023_1587_MOESM2_ESM.pptx]

## Slide 1
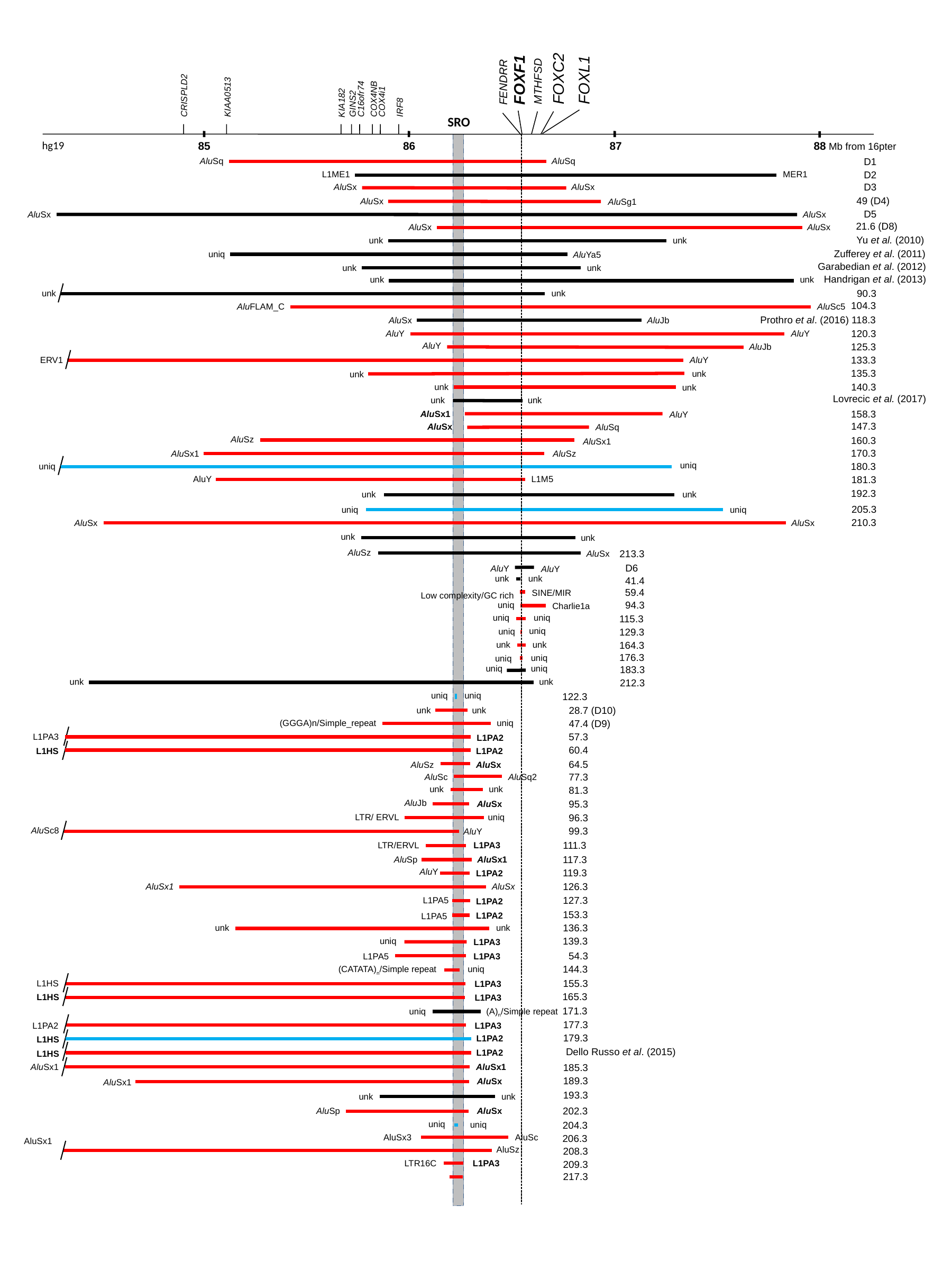

FOXC2
FOXF1
FOXL1
MTHFSD
FENDRR
CRISPLD2
KIAA0513
COX4NB
C16ofr74
COX4i1
KIA182
GINS2
IRF8
SRO
hg19
85
86
87
88
Mb from 16pter
AluSq
AluSq
D1
D2
L1ME1
MER1
D3
AluSx
AluSx
49 (D4)
AluSx
AluSg1
D5
AluSx
AluSx
21.6 (D8)
AluSx
AluSx
Yu et al. (2010)
unk
unk
Zufferey et al. (2011)
uniq
AluYa5
Garabedian et al. (2012)
unk
unk
Handrigan et al. (2013)
unk
unk
90.3
unk
unk
104.3
AluSc5
AluFLAM_C
Prothro et al. (2016) 118.3
AluSx
AluJb
120.3
AluY
AluY
AluY
125.3
AluJb
133.3
ERV1
AluY
135.3
unk
unk
140.3
unk
unk
Lovrecic et al. (2017)
unk
unk
158.3
AluSx1
AluY
147.3
AluSx
AluSq
AluSz
160.3
AluSx1
170.3
AluSx1
AluSz
uniq
180.3
uniq
181.3
AluY
L1M5
192.3
unk
unk
205.3
uniq
uniq
210.3
AluSx
AluSx
unk
unk
AluSz
213.3
AluSx
D6
AluY
AluY
unk
unk
41.4
59.4
SINE/MIR
Low complexity/GC rich
94.3
uniq
Charlie1a
uniq
uniq
115.3
uniq
129.3
uniq
unk
164.3
unk
176.3
uniq
uniq
uniq
uniq
183.3
unk
unk
212.3
uniq
uniq
122.3
28.7 (D10)
unk
unk
(GGGA)n/Simple_repeat
uniq
47.4 (D9)
57.3
L1PA3
L1PA2
60.4
L1HS
L1PA2
64.5
AluSz
AluSx
77.3
AluSc
AluSq2
unk
unk
81.3
AluJb
95.3
AluSx
LTR/ ERVL
uniq
96.3
AluSc8
99.3
AluY
111.3
LTR/ERVL
L1PA3
117.3
AluSx1
AluSp
AluY
119.3
L1PA2
126.3
AluSx1
AluSx
127.3
L1PA5
L1PA2
153.3
L1PA2
L1PA5
136.3
unk
unk
139.3
uniq
L1PA3
54.3
L1PA5
L1PA3
144.3
uniq
(CATATA)n/Simple repeat
155.3
L1HS
L1PA3
165.3
L1HS
L1PA3
171.3
uniq
(A)n/Simple repeat
177.3
L1PA2
L1PA3
179.3
L1PA2
L1HS
Dello Russo et al. (2015)
L1PA2
L1HS
AluSx1
AluSx1
185.3
189.3
AluSx
AluSx1
193.3
unk
unk
202.3
AluSp
AluSx
uniq
uniq
204.3
AluSc
AluSx3
206.3
AluSx1
AluSz
208.3
LTR16C
L1PA3
209.3
217.3
