# Supplementary material for: Further refinement of the differentially methylated distant lung-specific FOXF1 enhancer in a neonate with alveolar capillary dysplasia
Source: Clin Epigenetics. 2023 Oct 21;15:169. doi: 10.1186/s13148-023-01587-6 (PMC10589973; doi:10.1186/s13148-023-01587-6)
Supplement: Supplementary file 4 — Additional file 4: Figure S4. Parental origin of the CNV deletion. Based on SNV segregation, the deletion occurred on the maternal chr16. [file 13148_2023_1587_MOESM4_ESM.pptx]

## Slide 1
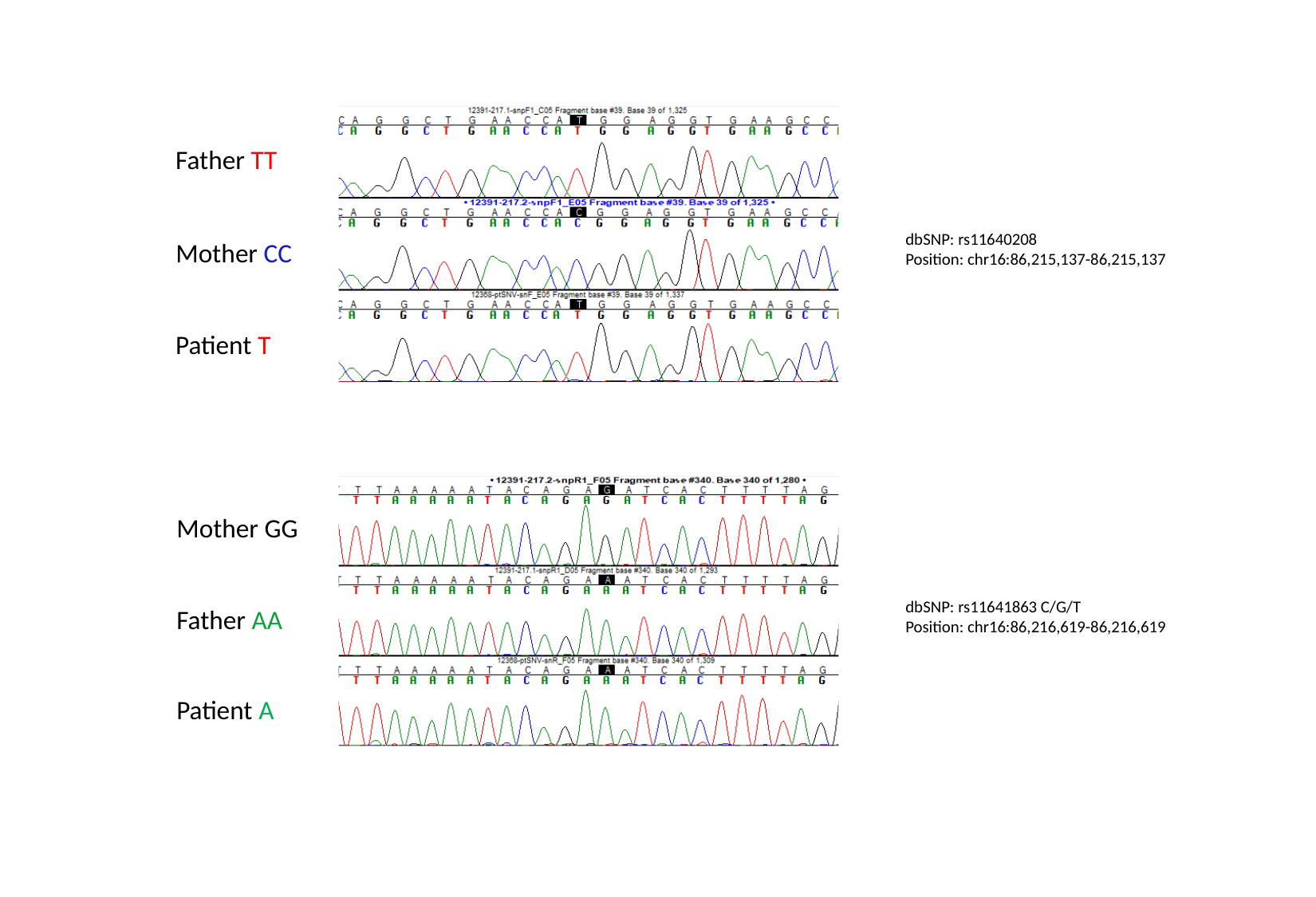

Father TT
dbSNP: rs11640208
Position: chr16:86,215,137-86,215,137
Mother CC
Patient T
Mother GG
dbSNP: rs11641863 C/G/T
Position: chr16:86,216,619-86,216,619
Father AA
Patient A
