# Supplementary material for: Further refinement of the differentially methylated distant lung-specific FOXF1 enhancer in a neonate with alveolar capillary dysplasia
Source: Clin Epigenetics. 2023 Oct 21;15:169. doi: 10.1186/s13148-023-01587-6 (PMC10589973; doi:10.1186/s13148-023-01587-6)
Supplement: Supplementary file 5 — Additional file 5: Figure S5. Relative FOXF1 and TMEM100 transcript levels in lungs with two partially overlapping FOXF1 enhancer deletions. [file 13148_2023_1587_MOESM5_ESM.pptx]

## Slide 1
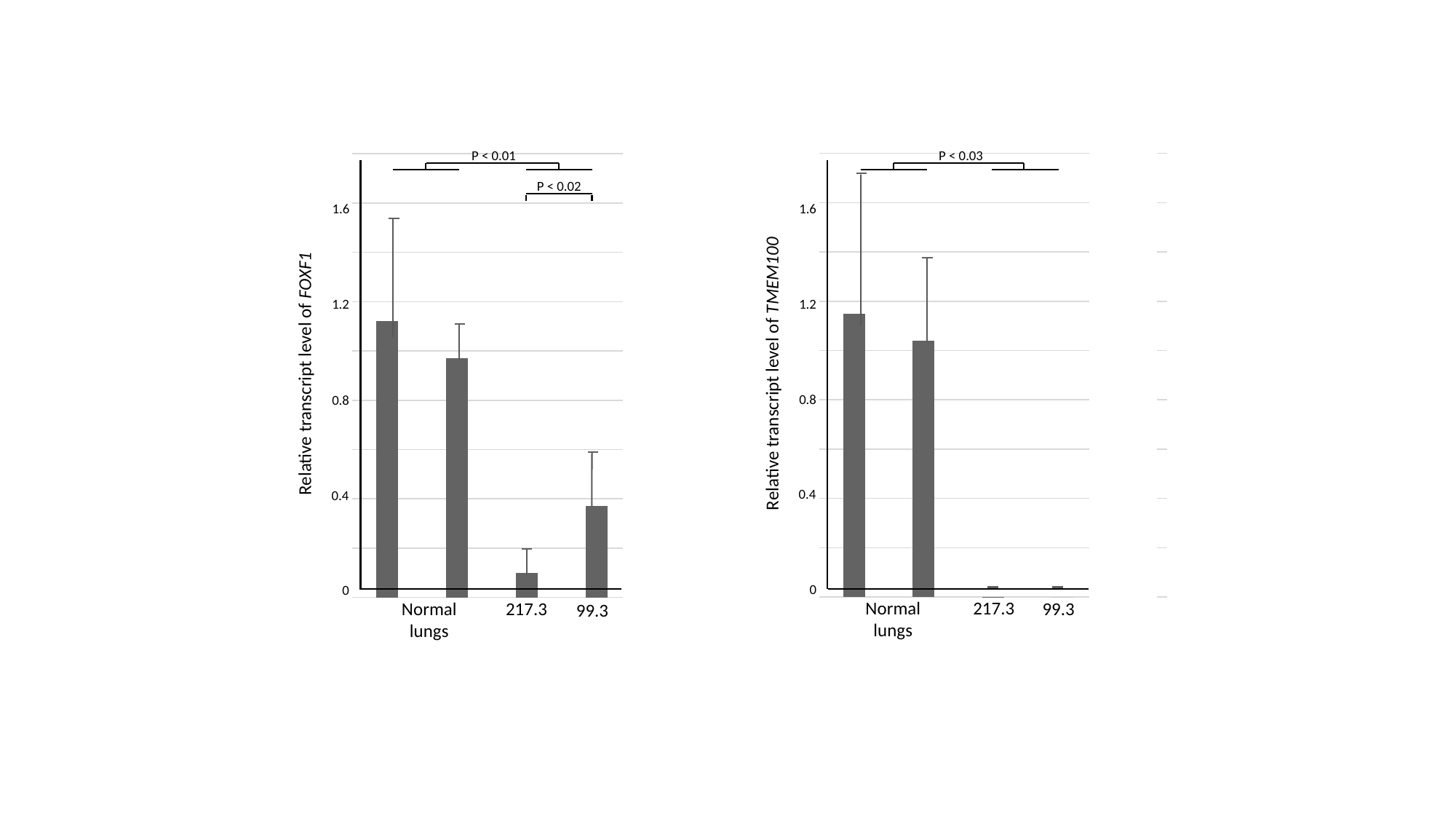

P < 0.01
### Chart
| Category | |
|---|---|
P < 0.02
1.6
1.2
Relative transcript level of FOXF1
0.8
0.4
0
Normal
lungs
217.3
99.3
P < 0.03
### Chart
| Category | |
|---|---|
1.6
1.2
Relative transcript level of TMEM100
0.8
0.4
0
Normal
lungs
217.3
99.3
