# Supplementary material for: Further refinement of the differentially methylated distant lung-specific FOXF1 enhancer in a neonate with alveolar capillary dysplasia
Source: Clin Epigenetics. 2023 Oct 21;15:169. doi: 10.1186/s13148-023-01587-6 (PMC10589973; doi:10.1186/s13148-023-01587-6)
Supplement: Supplementary file 8 — Additional file 8: Table S1. PCR primers used in methylation analysis. Table S2. Results of semi-quantitative analysis of DNA protection from MboI cleavage by adenine methylation in DMR regions of the enhancer Unit 2. The percentage values correspond to the extent of methylation and indicate on average higher methylation of Unit 2 on maternal chr16. [file 13148_2023_1587_MOESM8_ESM.docx]

| **Methylated DNA base** | **Enhancer region** | **PCR primer** |
| --- | --- | --- |
| Adenine (ApT) | DMR1 | GCCTCTCCTCACCTCAGTTTCCAAAT |
|  |  | TGGATGCTCGTTTTGTCCCATGAGT |
|  | DMR2 | GGCCATGGTCTCCTTTCTCTACAGT |
|  |  | GCTGTCCAACACTGAGCACCCTAG |
| Cytosine (CpG) | DMR1 | AGTCCTGCTCTCCTTGGGCAGT |
|  |  | ATCCTGCAGCATTTTCTTGGTGA |
|  | DMR2 | GGCCATGGTCTCCTTTCTCTACAGT |
|  |  | GCTGTCCAACACTGAGCACCCTAG |
|  | Internal control | AAGGGTCACTCCCTTTCATCCT |
|  |  | ACTCTGAGGTCCCACTGCTGAC |

**Supplementary Table S1**. PCR primers used in methylation analysis.

**Supplementary Table S2**. Results of semi-quantitative analysis of DNA protection from *Mbo*I cleavage by adenine methylation in DMR regions of the enhancer Unit 2. The percentage values correspond to the extent of methylation and indicate on average higher methylation of Unit 2 on maternal chr16.

| **Enhancer region** | **Pt 179.3** | **Pt 180.3** | **Pt 205.3** | **Average** | **Pt 60.4** | **Pt 64.5** | **Average** |
| --- | --- | --- | --- | --- | --- | --- | --- |
| Maternal chromosome 16 | | | | | Paternal chromosome 16 | | |
| DMR1 | 14% | 26% | 14% | 15.8±5.3% | 12% | 6% | 8.8±3.2% |
| DMR2 | 11% | 13% | 17% |  | 11% | 6% |  |
